# Crossover point of the field effect transistor and interconnect applications in turbostratic multilayer graphene nanoribbon channel

**DOI:** 10.1038/s41598-021-89709-z

**Published:** 2021-05-13

**Authors:** Ryota Negishi, Katsuma Yamamoto, Hirofumi Tanaka, Seyed Ali Mojtahedzadeh, Nobuya Mori, Yoshihiro Kobayashi

**Affiliations:** 1 Graduate School of Engineering, Osaka University, 2-1 Yamadaoka, Suita, Osaka 565-0871 Japan; 2 Research Center for Neuromorphic AI Hardware, Kyushu Institute of Technology, 2-1 Hibikino, Wakamatsu, Kitakyushu 808-0196 Japan; 3 Graduate School of Life Science and System Engineering, Kyushu Institute of Technology, 2-1 Hibikino, Wakamatsu, Kitakyushu, 808-0196 Japan; 4 Present Address: Faculty of Science and of Engineering, Department of Electrical, Electronics and Communications Engineering, Toyo University, 2100 Kujirai, Kawagoe, Saitama 350-8585 Japan

**Keywords:** Materials science, Nanoscience and technology

## Abstract

The electrical transport properties of a turbostratic multilayer graphene nanoribbon (GNR) with various number of layers (1–8 layers) were investigated using a field effect transistor with a single GNR channel. In the turbostratic multilayer GNR with 5 layers or less, the carrier mobility and I_on_/I_off_ ratio in the FETs were improved by slightly increasing the conductance with increasing the number of layers, meaning that the excellent semiconducting characteristic. The improvement of the carrier transport properties promotes by the turbostratic stacking structure. In the turbostratic multilayer GNR with 6 layers or more, although the I_on_/I_off_ ratio degraded, the conductance extremely improved with increasing the number of layers. This indicates that the turbostratic multilayer GNR with thicker number of layers becomes the significantly lower resistivity wire as a metallic characteristic. We revealed that the crossover point of the physical properties between the semiconducting and metallic characteristics is determined by the strength to screen the surrounding environment effects such as charged impurity on the substrate. Our comprehensive investigation provides a design guidance for the various electrical device applications of GNR materials.

## Introduction

Since theoretical calculation using a tight binding model predicted that a quasi-one dimensional monolayer graphene nanoribbon (GNR) exhibits a finite energy band gap at the Fermi level^1^, its anomalous electrical properties such as a band gap opening and electron confinement have been experimentally investigated in the GNR material^2–7^. The edge structures and the width of GNR material play an important role in its electrical band structures^8, 9^, and optical properties^10–13^. For example, the band gap width shrinks as the GNR width increases^14^. Therefore, the various synthesis methods of the GNR using electron beam lithography^15, 16^, chemical cutting or etching of graphene^17, 18^, nickel nanobars^19^ and precursor monomers as a bottom-up approach^7, 20^ have been studied for the precise control of the structures.

The monolayer or bilayer GNRs obtained by unzipping of single- or double-walled carbon nanotubes (CNTs) provide the narrow width and smooth edges^21, 22^, and the high I_on_/I_off_ ratio (10^5^–10^6^) and carrier mobility exceeding 100 cm^2^/Vs are demonstrated using the field effect transistors (FETs) with the single GNR channel^23, 24^. This result indicates that these GNRs have a semiconducting characteristic. On the other hand, the multilayer GNR can be synthesized by unzipping multi-walled CNTs^25, 26^ and the plasma-enhanced chemical vapor deposition (CVD) growth on nickel nanobars^27^. Recently, the demonstration of the extremely low resistivity in the metallic multilayer GNR^28^ had a great impact on the interconnect applications of the GNR materials^29–32^. The completely different physical properties such as semiconducting and metallic characteristics observed in the GNR materials are properly utilized in these device applications, respectively. However, there is no clear design guidelines for controlling the semiconducting and/or metallic characteristics in the real GNR devices except for the edge structure and GNR width in the ideal system treated in theory. What is the dominate factor that determines the conductive properties of the GNR material embedded in the device structure? The semiconducting and metallic characteristics tend to be mainly observed at a monolayer (or bilayer) GNR^6, 24^ and a multilayer GNR^28^, respectively. Therefore, we focus on the effect of the number of layers on the carrier transport properties of the GNR-FETs.

It is well known that the conductivity and carrier mobility of a monolayer graphene-FET extremely degrades on the SiO_2_/Si device substrate as a comparison with an intrinsic property of the monolayer graphene observed in a suspended structure^33^. Since the carrier concentration of the monolayer graphene with a linear dispersion is very low near the Dirac point, the carrier transport properties in the monolayer graphene-FETs are extremely sensitive to the surrounding environments such as the charged impurity and surface roughness^34^. On the other hand, in a multilayer graphene-FET, the carrier scattering induced by the surrounding environments is dramatically improved by the screening effect of the multi-stacking^35^. In the GNR system with a narrow width, the surrounding environment effect is considered to be extremely large compared with the graphene system.

In this study, to understand the surrounding environment effect on the carrier transport properties of the GNR, we investigated the transfer characteristics of the GNR-FETs with various number of layers (1–8 layers) on the SiO_2_/Si substrate. The turbostratic multilayer were synthesized by an overlayer growth of graphene on a GNR template prepared by unzipping from double-walled CNTs as previously reported^36^. We found that the carrier transport properties such as the semiconducting and metallic characteristics observed in the multilayer GNR with a narrow width (18–25 nm) are determined by strength to screen the charges of the impurity on the device substrate as a surrounding environment. This result indicates that the type of the device can be controlled by the number of layers in the multilayer GNR.

## Results

### Structural analysis of the multilayer GNRs

**Figure 1:**
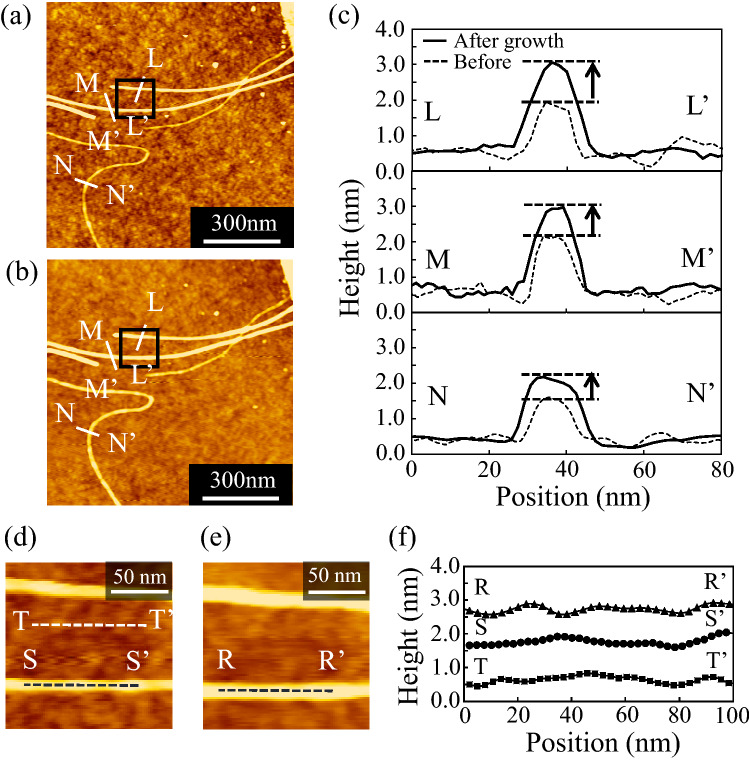
AFM images of GNRs (**a**) before and (**b**) after CVD growth, and (**c**) height profiles along L–L′, M–M′ and N–N′ lines, respectively. (**d**,**e**) are the enlarged AFM images obtained from the squares in (**a,b**). (**f**) is the surface roughness on the grown GNR and SiO_2_/Si substrate along S–S′, R–R′ and T–T′ lines, respectively. The AFM images are edited using a software AFM5000/AFM5010 Ver. 6.03A (C) Hitachi High-Tech Science Corp. 2013.

Figure 1a,b show the AFM images observed at the same location for the samples of the pristine and grown GNRs. Figure 1c is also the height profiles along L–L′, M–M′ and N–N′ lines, respectively. The height of the pristine GNR before the growth is ~ 1 nm. This value is obviously smaller than the diameter of the DWCNT (~ 3–15 nm)^22^ used as the starting material, and is slightly higher than the interlayer distance (0.34 nm) in the bulk graphite. Considering that the height of the monolayer graphene on the SiO_2_/Si substrate is 0.6–0.8 nm due to a very weak interaction between the graphene and substrate^37^, this result means that the monolayer or bilayer GNRs are efficiently synthesized by unzipping from the DWCNTs. After the CVD growth, the height of the GNRs are increased by ~ 1 nm, meaning the formation of additional two or three layers of graphene on the pristine GNR template. Figure 1d,e show the enlarged images as indicated by the square in Fig. 1a,b, respectively. The root mean square (RMS) surface roughness of the grown GNR and substrate evaluated from the height profiles along R–R′, S–S′ and T–T′ lines in Fig. 1f is almost the same value (0.10–0.11 nm) among them, and the value is lower than a monolayer graphene step height. This indicates that the surface morphology in the grown graphene layers is atomically flat.

**Figure 2:**
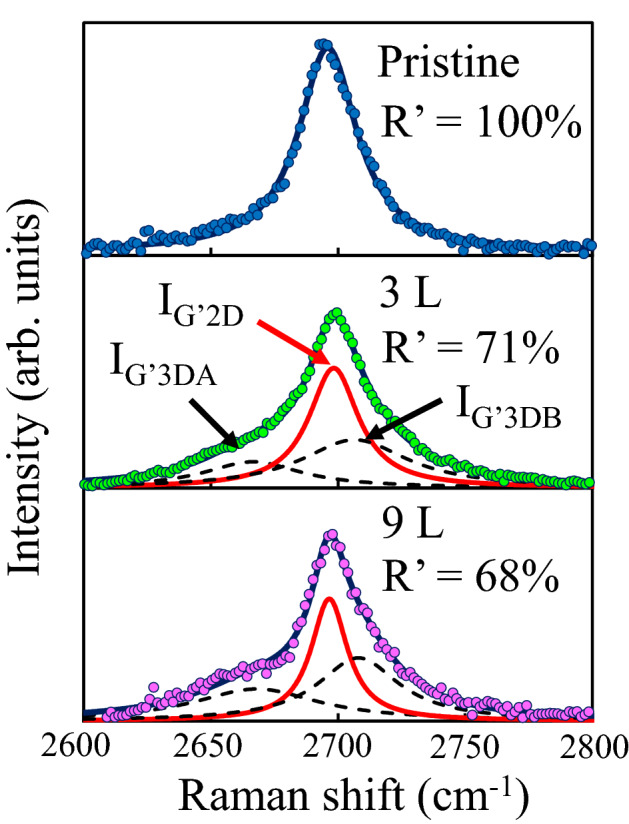
Raman spectra in the 2D-band region observed from the pristine monolayer and grown multilayer GNR. The dotted circles and thin solid lines are the experimental data and fitting curves, respectively.

The Raman spectrum in the 2D-band region provides an evaluation method to distinguish the different number of layers and stacking structures in multilayer graphene system^38^. Figure 2 shows the 2D-band spectra observed from the pristine and grown GNR with different number of layers. The number of layers in the GNR is decided by the AFM observations. The dotted circles and thin solid lines indicate the experimental data and the fitting curves, respectively. The spectrum is normalized by the integrated intensities of the 2D-band region from 2600 to 2800 cm^−1^. The spectrum observed from the pristine GNR can be fitted by a single Lorentzian peak, indicating that the pristine GNR is constructed by a monolayer structure. The stacking structures in the grown multilayer graphene are mainly divided into two types: the ordered stacking (e.g. AB stacking) and turbostratic stacking. In a multilayer graphene with the ordered stacking, the peak shape in the 2D-band spectrum is decomposed by two Lorentzian peaks at ~ 2680 and ~ 2720 cm^−1^, respectively^39^. On the other hand, the peak shape observed from a turbostratic multilayer graphene, in which the stacking of the graphene layers is rotationally random in plane, shows a single Lorentzian at ~ 2700 cm^−1^, just as in monolayer graphene^40^. Therefore, the turbostratic ratio (R′) in the multilayer graphene can be evaluated by the curve fitting analysis of the Raman spectrum in the 2D-band region using three Lorenz curves as the peak positions at ~ 2680, ~ 2700 and ~ 2720 cm^−1^, as noted by I_G’3DA_, I_G’2D_ and I_G’3DB_ using the following equation^41^:

1 $${\text{R}}^{\prime} = 1 - {\text{R = 1}} - \frac{{{\text{I}}_{{{\text{G}}^{\prime}3{\text{DB}}}} }}{{{\text{I}}_{{{\text{G}}^{\prime}3{\text{DB}}}} + {\text{I}}_{{{\text{G}}^{\prime}2{\text{D}}}} }},$$

where the R means the ordered stacking ratio. The ratios of turbostratic stacking in the pristine graphene and the grown multilayer graphene with 3 and 9 layers are about 100%, 71% and 68%, indicating that the grown graphene layer forms a turbostratic stacking at a high ratio.

### Electrical transport properties of the turbostratic multilayer GNR

**Figure 3:**
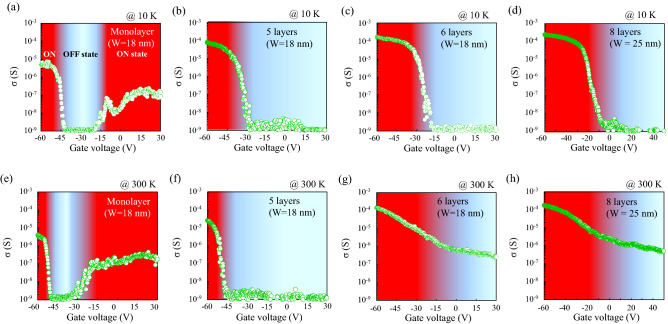
Transfer characteristics in the (**a**,**e**) monolayer and (**b**–**d**,**f**–**h**) grown multilayer GNR-FETs measured at 10 K and 300 K, respectively.

Figure 3 shows the transfer characteristics (conductance vs gate voltage) in the FETs using a single channel of the pristine GNR and the grown multilayer GNR with various number of layers measured at (a)–(d) 10 K and (e)–(h) 300 K, respectively. The conductance (σ) is defined as the inverse of the sheet resistance (R_s_) as the following equation:

2 $${\sigma } = { }\frac{1}{{{\text{R}}_{{\text{s}}} }}{ } = { }\left( {\frac{{{\text{I}}_{{{\text{sd}}}} }}{{{\text{V}}_{{{\text{sd}}}} }}} \right)\left( {\frac{{{\text{L}}_{{{\text{ch}}}} }}{{{\text{W}}_{{{\text{ch}}}} }}} \right),$$

where I_sd_ and V_sd_ are the source-drain current and source-drain voltage, respectively. L_ch_ and W_ch_ are also the length and width of the single GNR channel in the FET, respectively. Although the width of the GNR tends to increase with the growth (see Supplementary Fig. S1), the W_ch_ measured in the transfer characteristics is almost the same value (18–25 nm) among them as indicated by the inset in Fig. 3. At the low temperature, the minimum value of conductance (σ_min_) as an OFF state region reaches the detection limit value of current (~ 10 pA) in our source-measure unit, and the semiconducting characteristics are observed. The strong suppression of conductance in the OFF state region is caused by the formation of the transport gaps associated with the disordered structures^42^. On the other hand, at room temperature, the transfer characteristics measured at the grown multilayer GNR with 6 layers or more slightly depends on the temperature, resulting the degradation of I_on_/I_off_ ratio. This means that the suppression effect in the grown multilayer GNR significantly reduces with increasing measurement temperature (the details of the carrier transport mechanism will be described in the section of “Discussion”).

**Figure 4:**
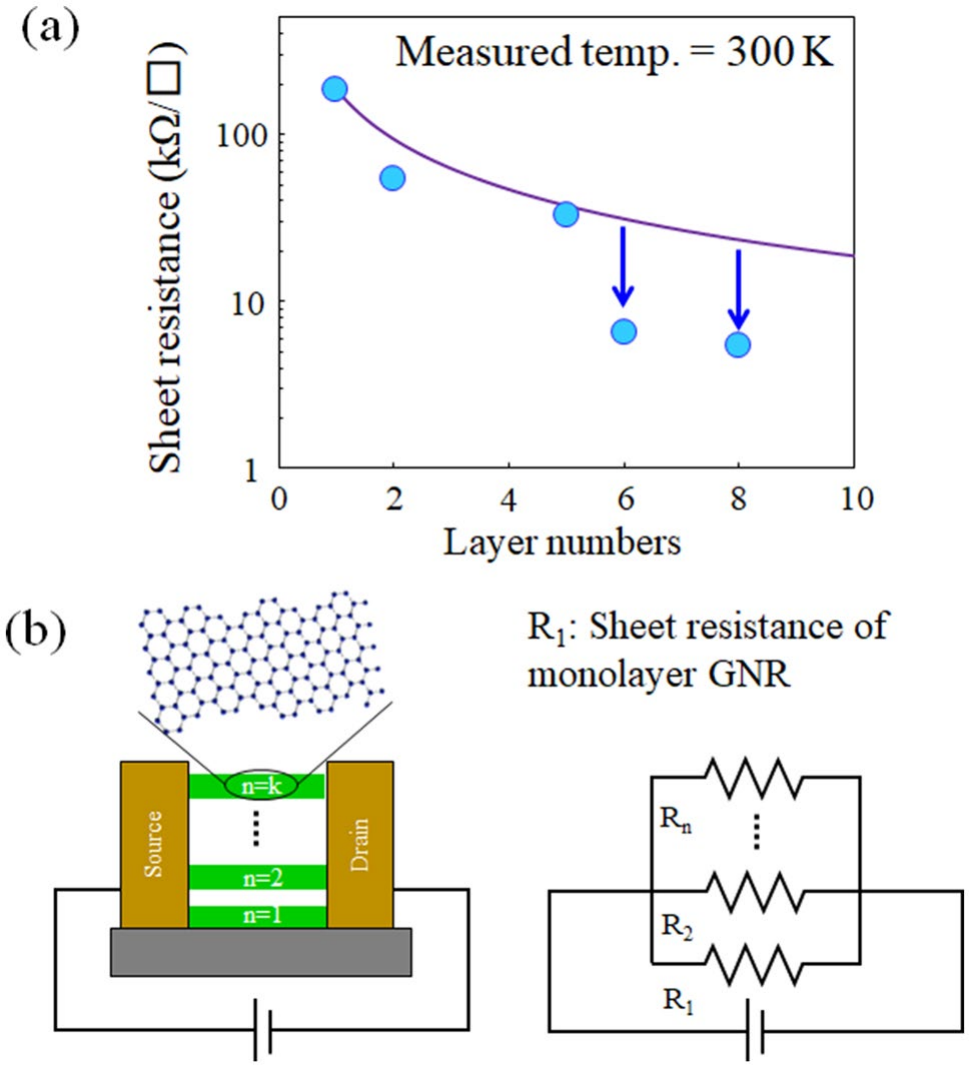
(**a**) Sheet resistance measured at ON state region vs the number of layers in the GNR-FET. (**b**) Is a resistance model composed of parallel channel, assuming that the conductance between layers can be ignored.

Next, we focus on the sheet resistance corresponding the maximum conductance (σ_max_) value at the ON state region. Figure 4 is the change in the sheet resistance as a function of the number of layers. Note that the sheet resistance decreases with increasing the number of layers. We consider a simple resistance model in which the plane direction resistance of the layers is connected in parallel between the source and the drain electrodes, assuming that the conductance between graphene layers connected by van der Waals force can be ignored as shown in Fig. 4b. Then, the total sheet resistance (${\text{R}}_{{{\text{total}}}}$) of the multilayer GNR composed of n layers is described as

3 $$\frac{{1}}{{{\text{R}}_{{{\text{total}}}} }}{ = }\frac{{1}}{{{\text{R}}_{{1}} }}{ + }\frac{{1}}{{{\text{R}}_{{2}} }}{ + } \cdots { + }\frac{{1}}{{{\text{R}}_{{\text{n}}} }},$$

where R_n_ and R_1_ are the sheet resistance of monolayer GNR in the n-th layer and pristine monolayer GNR as a growth template. Assuming that the sheet resistance of the grown monolayer GNR in each layer is equal to that of the pristine monolayer GNR as a growth template, the change in the sheet resistance with respect to the number of layers evaluated from the calculation becomes to be a curve as indicated by the solid line in Fig. 4a. However, the observed resistance value deviates from the calculated curve and show a lower resistance as indicated by arrows. The same behavior of the significant improvement in conductance with increasing the number of layers has been also observed in the turbostratic multilayer graphene^43, 44^. The improvement is explained by reducing the carrier scattering due to the screening effect of the multi-stacking for the charges of the impurity on the SiO_2_/Si substrate (the detailed mechanism of the improvement in conductance will be described in the “Discussion” section). A notable difference between the multilayer graphene and multilayer GNR is the reduction rate of the sheet resistance as the number of layer increases. In the multilayer graphene, the sheet resistance of 7-layer graphene is ~ 0.7 times higher than that of single layer graphene^44^. In contrast, the sheet resistance of 6 or 8-layer GNR is ~ 0.3 times higher than that of single layer GNR (see Fig. 4a), indicating a larger reduction rate. The difference of the reduction rate of the sheet resistance between the graphene and GNR is thought to be caused by the structural dimensionality. In the multilayer GNR-FET, the channel width is extremely narrow due to a one-dimensional structure, and the effect of the carrier scattering by the charge impurities existed on the SiO_2_/Si substrate is significant. As a result, the conductance enhancement by the screening effect in the multilayer GNR is expected to be more pronounced than that in the multilayer graphene as a two-dimensional system.

**Figure 5:**
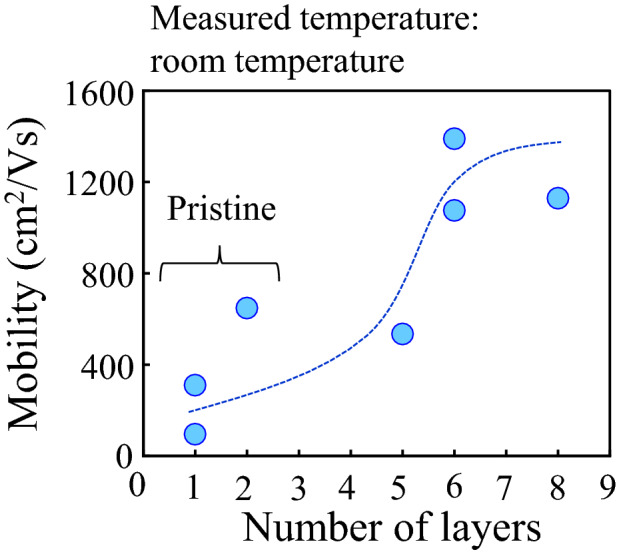
Field effect mobility vs the number of layers measured at room temperature. The monolayer and bilayer GNR is a pristine GNR. The dashed line is an eye guide.

Figure 5 shows the carrier mobility of the GNR as a function of the number of layers measured at room temperature. The circles and dashed line are experiment data and eye guide, respectively. The pristine GNRs prepared by unzipping from the DWCNTs are composed of monolayer or bilayer. The carrier mobility observed from the pristine GNR is in good agreement with the value as previously reported^23^. Note that the carrier mobility significantly improves with increasing the number of layers. Usually, the carrier mobility observed from a few layer graphene composed of the AB (Bernal) stacking structure degrades with increasing the number of layers because the electrical band structure near the Dirac point approaches a parabolic dispersion^35, 45^. It has been reported that the carrier mobility observed in a few layer graphene with the turbostratic stacking structure is higher than that of the monolayer graphene^46^ because the electrical band structure keeps the linear dispersion due to a weak inter-layer coupling between the layers^40^. Therefore, it is considered that the anomalous carrier transport properties in the turbostratic multilayer GNR as a function of the number of layers is caused by the improvement of transfer conductance associated with keeping the linear dispersion and the screening effect.

## Discussion

**Figure 6:**
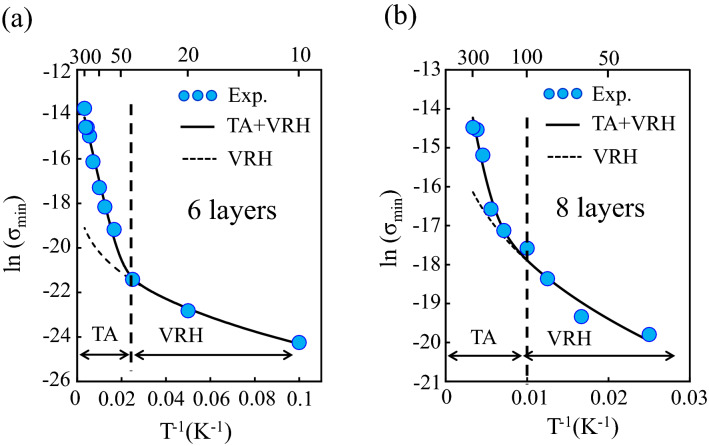
A plot of ln (σ_min_) measured from the multilayer GNR with (**a**) 6 and (**b**) 8 layers vs T^−1^, respectively. The circles and solid lines are experimental data and the fitting curves by the sum of the TA and 2D-VRH conductions.

We discuss the carrier transport mechanism of the GNR under the gate bias region exhibiting a minimum conductance (σ_min_) value as an OFF state. As shown in Fig. 3, the σ_min_ of the multilayer GNR with 6 layers or more at the OFF state region depends on the measurement temperature. The analysis of temperature dependence in conductance is a convenient way to identify the dominate factors of such carrier scattering as phonons and charged impurities^47^. Figure 6 shows a plot of ln (σ_min_) as a function of T^−1^. The temperature dependence of ln (σ_min_) measured from the grown multilayer GNRs with 6 and 8 layers is well fitted by the sum of the two-dimensional variable range hopping (2D-VRH) and thermal activation (TA) conductions^48^, expressed by

4 $${\upsigma }_{{{\text{min}}}} {\text{ (T) }} \,= \sigma _{{{\text{VRH}}}} {\text{ (T)}} + \sigma _{{{\text{TA}}}} {\text{ (T),}}$$

where the σ_VRH_ (T) and σ_TA_ (T) are the temperature dependence of conductance based on the 2D-VRH and TA conduction models, respectively. In the 2D-VRH model, the σ_VRH_ (T) has the following formula^49^

5 $${\upsigma }_{{{\text{VHR}}}} \left( {\text{T}} \right){ } \propto {\text{ exp}}\left( {{\text{B/T}}^{{{1}/{3}}} } \right),$$

where B is hopping parameter. The carriers in the 2D-VRH conduction thermally jump from one localized state to another between a large energy gap without going through a continuous electrical band structure. The phenomenon is frequently observed in reduced graphene oxide materials with many defects^50, 51^. In the TA conduction model, the σ_TA_ (T) is expressed by

6 $${\upsigma }_{{{\text{TA}}}} {\text{ (T) }} \propto {\text{ exp}}\left( {-{\text{ E}}_{{\text{a}}} /{\text{k}}_{{\text{B}}} {\text{T}}} \right),$$

where the E_a_ and k_B_ are the thermal activation energy and the Boltzmann constant, respectively. The TA conduction means that the carriers flow via a continuous electrical band structure. When the measurement temperature rises at a boundary of around 40–100 K as indicated by the vertical dashed lines in Fig. 6, the conduction mechanism in the grown multilayer GNR with 6 and 8 layers changes from the 2D-VRH to the TA conductions. This means that the suppression effect of conduction significantly reduces due to increasing the measurement temperature. The σ_min_ in the pristine GNR and the grown multilayer GNR with 5 layers or less is below the detection limit value at room temperature (see Fig. 3), meaning that the conduction mechanism is dominated by the 2D-VRH conduction due to the strong suppression of the carrier transport even at room temperature.

We focus on the origin of reducing the suppression effect in the grown multilayer GNR with 6 and 8 layers. The suppression effect of the carrier transport is induced by the transport gap that arises from quantum confinement and edge effects^1, 52^. The suppression is extremely enhanced at the Dirac point where the carrier concentration shows a minimum value due to the charge neutrality point. As a result, the conductance value of the monolayer GNR around the Dirac point sometimes exhibits zero-conductance as an OFF state^4^. The theoretical calculation predicts that a GNR with the width of 5 nm or less is required for the formation of the band gap energy larger than room temperature^53^ although the band gap energy also depends on the edge structures. However, the transport gaps have been experimentally observed even with lager widths (~ 25 nm)^4, 54^. Moreover, we observe that the strength of the suppression effect differs greatly between the pristine monolayer GNR and the grown multilayer GNR, despite the fact that the width is almost the same between them (see Supplementary Fig. S2). These mean that the experimentally observed value of the transport gap associated with electron confinement in the GNR system is not determined only by the width and edge structures. Usually, there are many charged impurities (~ 10^12^ cm^−2^) on the surface of the SiO_2_/Si device substrate. Since the amount of charged impurities on the device substrate is larger than a typical carrier concentration (~ 10^11^ cm^−2^) of monolayer graphene near the Dirac point, the conductance and the carrier mobility significantly degrade on the device substrate due to the carrier scattering by the charged impurities^55, 56^. The similar situation applies to the GNR system. Since the charged impurities induce the localized potential barriers in the GNR with a narrow width, it is considered that the transport gap is significantly induced by the charged impurities.

The appearance temperature of the TA conduction is expected to increase with increasing the number of layer. However, the opposite trend is observed as shown in Fig. 6. Both 6- and 8-layer GNR-FETs are thought to have efficiently screening effect, considering that the screening length in the multilayer graphene is to be ~ 1 nm^57^. Therefore, the temperature range of the TA conduction in the multilayer GNR with a thickness of more than 1 nm seems to be strongly dependent on the crystallinity of the synthesized GNR rather than the number of layers. In fact, a similar carrier transport mechanism with the combination of TA and VRH conductions has been observed in the reduced graphene oxide material^48^, and the appearance temperature of the TA conduction strongly depends on the crystallinity. In the synthesis of multilayer graphene grown on graphene template via our CVD process, since the crystallinity tends to decrease as the number of layer increases^43^, it seems that the appearance of the TA conduction is observed at higher temperature in the 6-layer GNR-FET than the 8-layer GNR-FET.

**Figure 7:**
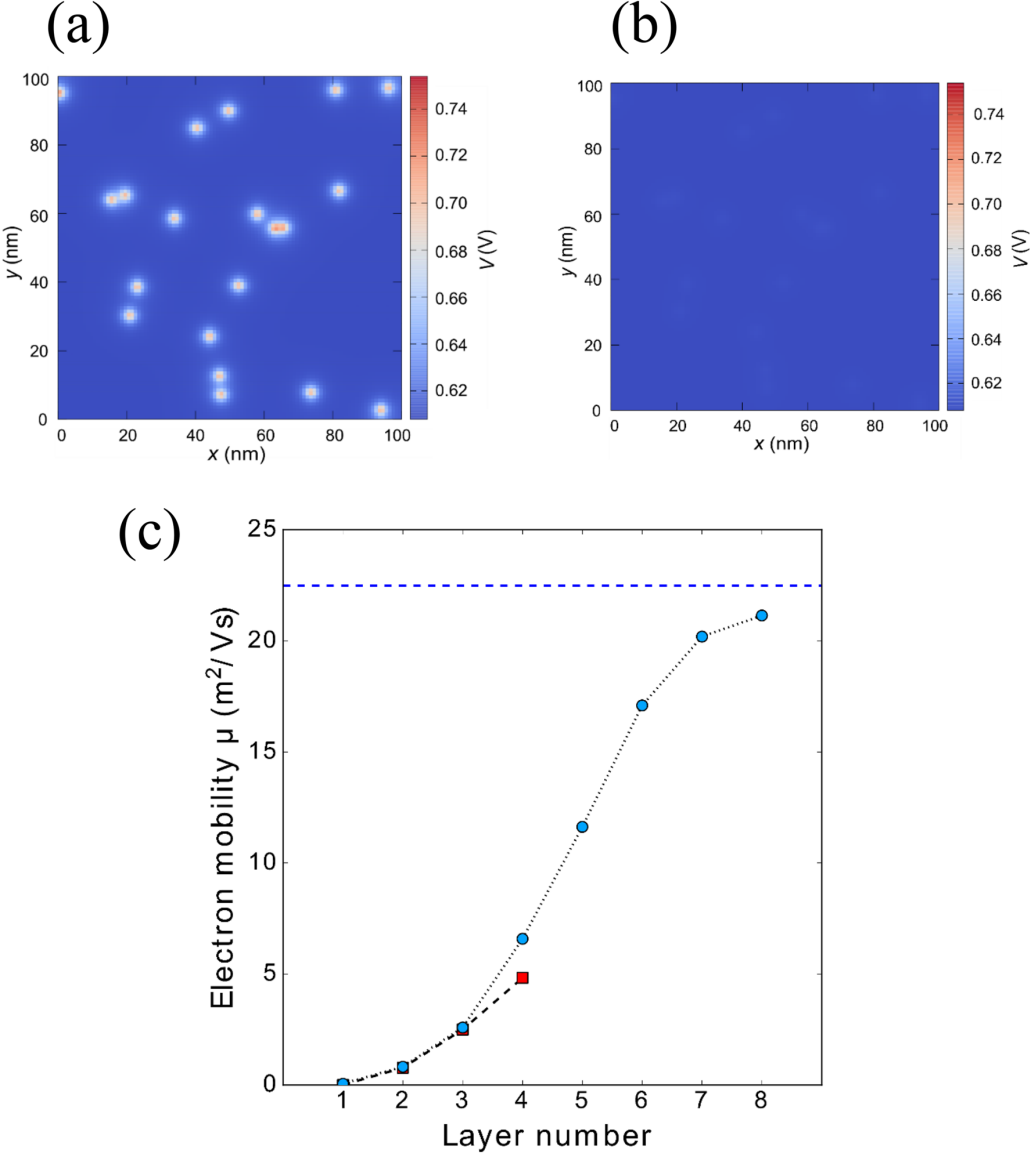
Self-consistent potential profiles at (**a**) the bottom layer and (**b**) the top layer in the multilayer GNR composed of 8 layers on the SiO_2_ substrate when the density of charged impurity is 2 × 10^11^ cm^−2^. (**c**) Is calculated electron mobility vs the number of layers. The multilayer GNR channels with 4 and 8 layers are described by red squares and blue circles, respectively.

We discuss the effect of the charged impurities on the carrier transport in the turbostratic multilayer GNR. Figure 7a,b show the self-consistent potential profiles of the bottom layer and the top layer in the multilayer GNR with 8 layers on the SiO_2_ substrate when the density of charged impurity is 2 × 10^11^ cm^−2^. The potential profile is calculated within the Thomas–Fermi approximation. In the bottom layer attached on the substrate, the surface potential is strongly modulated by the charged impurities, indicating that the electron–hole puddles as the localized states near the charge neutrality point are formed by the potential barrier of the charged impurities^58^. In this case, the carrier transport property is dominated by the 2D-VRH conduction via the localized states. On the other hand, the modulation of the surface potential due to the charged impurities dramatically reduces in the top layer because the charges on the substrate is screened in the interlayer of the multilayer GNR. Since the electrical band structure in the top layer of the multilayer GNR is not modulated by the charged impurities, the carrier transport is dominated by the TA conduction via a continuous band structure without any localized states as indicated by Fig. 6. The same screening effect are also observed at the multilayer graphene^35, 57^. As a result, the significant improvement of the conductance in the turbostratic multilayer GNR is observed by the efficient screening effect as shown in Fig. 4a.

**Figure 8:**
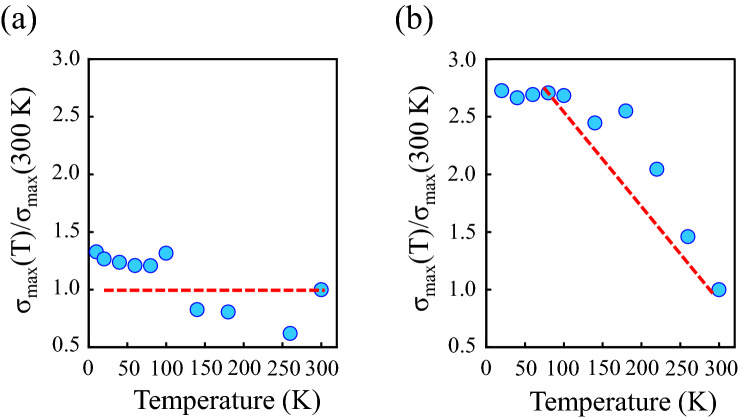
Temperature dependence of the σ_max_ measured from (**a**) the pristine monolayer GNR-FET and (**b**) the grown multilayer (6L) GNR-FET, respectively.

Figure 7c shows the calculated carrier mobility in each layer of the turbostratic multilayer GNR with 4 layers (squares) and 8 layers (circles). The carrier mobility improves with increasing the number of layers due to the enhancement of the screening effect. The improvement of the mobility becomes apparent in the vicinity of 4th to 6th layer, and this trend is in good agreement with the experiment result as shown in Fig. 5. It has been reported that the screening length in multilayer graphene is ~ 1 nm^57, 59^. In the simulation, the mobility is asymptotic to the value calculated by a model considering only phonon scattering, as indicated by the dashed line. Figure 8 shows the temperature dependence of the maximum conductance (σ_max_ (T)) in the pristine monolayer and the grown multilayer GNR-FET as the ON state region. The σ_max_ (T) is normalized by the σ_max_ (300 K) measured at room temperature. For the pristine monolayer GNR-FET, the σ_max_ (T) is independent to the measurement temperature. It has been reported that the conductance observed from the monolayer graphene is independent to the measurement temperature because the density of the charged impurities on the substrate is larger than the thermally excited carriers around the Dirac point due to the linear dispersion^35^. The similar situation applies to the GNR system. As a result, the conductance of the pristine GNR shows a lower value due to the strong suppression of the carrier transport by the localized potential barriers of the charged impurities as indicated by Fig. 3. Note that σ_max_ (T) in the grown multilayer GNR-FET significantly improves as the measurement temperature decreases as shown in Fig. 8b. The improvement of the σ_max_ (T) is caused by reducing the phonon scattering. Since the carrier scattering of the charged impurities is greatly reduced by the screening effect of the multi-stacking, the dominate factor of the carrier scattering is phonon scattering. This leads to the anomalous enhancements of the conductivity and carrier mobility as shown in Figs. 4 and 5.

**Figure 9:**
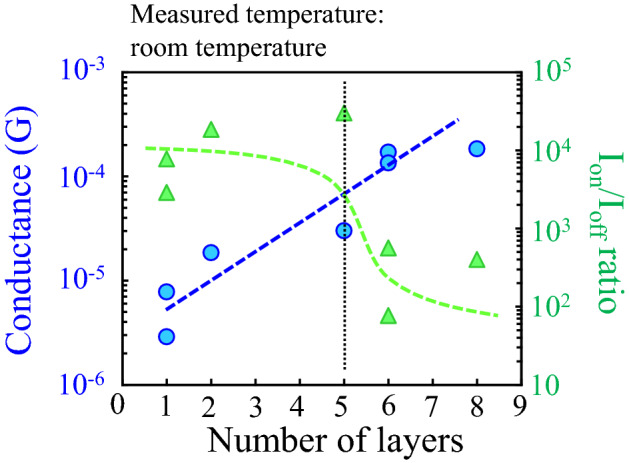
Overview of the device performance in the pristine and grown multilayer GNR-FETs measured at room temperature.

Figure 9 shows an overview of the device characteristics in the turbostratic multilayer GNR-FET measured at room temperature. The device characteristics greatly change in the vicinity of the 5th layer as a crossover point. At the turbostratic multilayer GNR with 5 or less layers, the zero-conductance state as a transport gap keeps even at room temperature due to a strong suppression effect of the carrier transport by the charged impurities on the substrate. Moreover the I_on_/I_off_ ratio and carrier mobility in the GNR-FET are improved with increasing the additional graphene layers with the turbostratic stacking. As a result, the transfer characteristic in the turbostratic multilayer GNR exhibits the excellent semiconducting property. When the grown multilayer GNR is 6 layers or more, the suppression of the conductance leading to the carrier scattering dramatically reduces by the efficient screening effect of the multi-stacking. As a result, the transfer characteristic in the multilayer graphene shows the metallic property with the significantly higher conductance without the transport gap.

## Summary

We investigated the carrier transport properties of the turbostratic multilayer GNR with various number of layers. At the grown multilayer GNR with a thin film thickness of 5 layers or less, the excellent I_on_/I_off_ ratio and carrier mobility were observed due to the suppression of the carrier transport and the turbostratic effect. When the number of layers exceeds 6 layers, the conductance at the ON state region extremely enhances by reducing the carrier scattering due to the screening effect for the charges of the impurity on the substrate. We conclude that the semiconducting and metallic characteristics in the narrow width GNR system can be controlled by modulating the strength of the screening effect to the surrounding environment such as charged impurity.

## Methods

### Synthesis of the multilayer GNR

The pristine GNRs used as a growth template were synthesized by unzipping of double-walled CNTs (Tokyo Ohka Kogyo Co. Ltd.)^22^. The synthesized GNRs were dispersed on the SiO_2_ (300 nm)/Si substrate, and the samples were preheated at 350 °C for 20 min in air under atmospheric pressure to remove the poly (*m*-phenylenevinylene-co-2,5-dioctoxy-*p*-phenylenevinylene (PmPV) used as a surfactant for the stabilization of unzipped GNRs in the solution. Subsequently, the graphene layers were grown on the GNR template using a sloped-temperature CVD apparatus with ethanol^60, 61^. The CVD apparatus can regulate the temperatures separately in three zones, resulting in individual control over the decomposition reaction of the carbon feedstock and the growth of graphene layers by activated carbon species. The temperatures used in this study were 900 °C and 720–744 °C for the decomposition and graphene layer growth, respectively.

### Structural and electrical analysis of the multilayer GNR

The structural analysis of the synthesized GNR was performed by atomic force microscope (AFM: HITACHI AFM5100N) observations with the dynamic force mode and Raman spectroscopy (Horiba HR800UV) with a laser excitation of 532 nm (typical spot diameter is 1 μm). The GNR-FET devices were fabricated by a conventional photolithographic process. The electrical transport measurements were carried out for the FET devices with just only single GNR channel. The carrier mobility were evaluated from the source-drain current (I_sd_) as a function of the gate voltage (V_g_) in the FET using a standard formula^62–64^, expressed by

7 $${{\upmu = }}\left( {\frac{{{\text{L}}_{{{\text{ch}}}} }}{{{\text{W}}_{{{\text{ch}}}} }}} \right)\left( {\frac{{1}}{{{\text{V}}_{{{\text{sd}}}} }}} \right)\left( {\frac{{1}}{{{\text{C}}_{{\text{g}}} }}} \right)\left( {\frac{{{\text{dI}}_{{{\text{sd}}}} }}{{{\text{dV}}_{{\text{g}}} }}} \right),$$

where W_ch_, L_ch_ and C_g_ are the width of the GNR, the channel length and the gate capacitance, respectively. The gate capacitance is given by C_g_ = ε_i_ε_0_/d. The ε_i_ and ε_0_ are the relative permittivity of SiO_2_ and the permittivity of a vacuum, and d is the film thickness of the SiO_2_ (300 nm).

### Simulation method

The electron mobility in a turbostratic multilayer GNR on the SiO_2_ substrate with a rectangle shape of L_ch_ × W_ch_ was calculated using a semiclassical Monte Carlo approach considering the surface phonon scattering and the charged impurity scattering induced by the substrate^65, 66^. The gapless energy dispersion at K_1_ and K_2_ points was used as the band structure because the inter-layer coupling interaction between GNR sheets to mimic the turbostratic stacking in the multilayer GNR.

## Supplementary Information

Supplementary Information.

## References

[1] Nakada K, Fujita M, Dresselhaus G, Dresselhaus MS (1996). Edge state in graphene ribbons: Nanometer size effect and edge shape dependence. Phys. Rev. B.

[2] Wagner P (2013). Band gap engineering via edge-functionalization of graphene nanoribbons. J. Phys. Chem. C.

[3] Li G (2018). A modular synthetic approach for band-gap engineering of armchair graphene nanoribbons. Nat. Commun..

[4] Han MY, Brant JC, Kim P (2010). Electron transport in disordered graphene nanoribbons. Phys. Rev. Lett..

[5] Lee G (2020). Transport gaps in ideal zigzag-edge graphene nanoribbons with chemical edge disorder. Appl. Surf. Sci..

[6] Wang XR (2011). Graphene nanoribbons with smooth edges behave as quantum wires. Nat. Nanotechnol..

[7] Slota M (2018). Magnetic edge states and coherent manipulation of graphene nanoribbons. Nature.

[8] Fujita M, Wakabayashi K, Nakada K, Kusakabe K (1996). Peculiar localized state at zigzag graphite edge. J. Phys. Soc. Jpn..

[9] Wakabayashi K, Fujita M, Ajiki H, Sigrist M (1999). Electronic and magnetic properties of nanographite ribbons. Phys. Rev. B.

[10] Chung HC, Chang CP, Lin CY, Lin MF (2016). Electronic and optical properties of graphene nanoribbons in external fields. Phys. Chem. Chem. Phys..

[11] Chung HC, Lee MH, Chang CP, Lin MF (2011). Exploration of edge-dependent optical selection rules for graphene nanoribbons. Opt. Express.

[12] Sasaki K, Kato K, Tokura Y, Oguri K, Sogawa T (2011). Theory of optical transitions in graphene nanoribbons. Phys. Rev. B..

[13] Hsu H, Reichl LE (2007). Selection rule for the optical absorption of graphene nanoribbons. Phys. Rev. B.

[14] Nakada K, Fujita M, Wakabayashi K, Kusakabe K (1996). Localized electronic states on graphite edge. Czech J. Phys..

[15] Meng N, Fernandez JF, Vignaud D, Dambrine G, Happy H (2011). Fabrication and characterization of an epitaxial graphene nanoribbon-based field-effect transistor. IEEE Trans. Electron Devices.

[16] Sommer B (2015). Electron-beam induced nano-etching of suspended graphene. Sci. Rep..

[17] Sun J, Iwasaki T, Muruganathan M, Mizuta H (2015). Lateral plasma etching enhanced on/off ratio in graphene nanoribbon field-effect transistor. Appl. Phys. Lett..

[18] Datta SS, Strachan DR, Khamis SM, Johnson ATC (2008). Crystallographic etching of few-layer graphene. Nano Lett..

[19] Kato T, Hatakeyama R (2012). Site- and alignment-controlled growth of graphene nanoribbons from nickel nanobars. Nat. Nanotechnol..

[20] Cai JM (2010). Atomically precise bottom-up fabrication of graphene nanoribbons. Nature.

[21] Jiao LY, Zhang L, Wang XR, Diankov G, Dai HJ (2009). Narrow graphene nanoribbons from carbon nanotubes. Nature.

[22] Tanaka H (2015). Method for controlling electrical properties of single-layer graphene nanoribbons via adsorbed planar molecular nanoparticles. Sci. Rep..

[23] Li XL, Wang XR, Zhang L, Lee SW, Dai HJ (2008). Chemically derived, ultrasmooth graphene nanoribbon semiconductors. Science.

[24] Wang XR (2008). Room-temperature all-semiconducting sub-10-nm graphene nanoribbon field-effect transistors. Phys. Rev. Lett..

[25] Dhakate SR, Chauhan N, Sharma S, Mathur RB (2011). The production of multi-layer graphene nanoribbons from thermally reduced unzipped multi-walled carbon nanotubes. Carbon.

[26] Elias AL (2010). Longitudinal cutting of pure and doped carbon nanotubes to form graphitic nanoribbons using metal clusters as nanoscalpels. Nano Lett..

[27] Suzuki H (2016). Wafer-scale fabrication and growth dynamics of suspended graphene nanoribbon arrays. Nat. Commun..

[28] Jiang JK (2017). Intercalation doped multilayer-graphene-nanoribbons for next-generation interconnects. Nano Lett..

[29] Hazra A, Basu S (2018). Graphene nanoribbon as potential on-chip interconnect material—A review. J. Carbon Res..

[30] Zhao WS, Yin WY (2014). Comparative study on multilayer graphene nanoribbon (MLGNR) interconnects. IEEE Trans. Electromagn. Compat..

[31] Xu CA, Li H, Banerjee K (2009). Modeling, analysis, and design of graphene nano-ribbon interconnects. IEEE Trans. Electron Devices.

[32] Hamedani SG, Moaiyeri MH (2020). Comparative analysis of the crosstalk effects in multilayer graphene nanoribbon and MWCNT interconnects in sub-10 nm technologies. IEEE Trans. Electromagn. Compat..

[33] Bolotin KI (2008). Ultrahigh electron mobility in suspended graphene. Solid State Commun..

[34] Tan YW (2007). Measurement of scattering rate and minimum conductivity in graphene. Phys. Rev. Lett..

[35] Nagashio K, Nishimura T, Kita K, Toriumi A (2010). Systematic investigation of the intrinsic channel properties and contact resistance of monolayer and multilayer graphene field-effect transistor. Jpn. J. Appl. Phys..

[36] Negishi R (2017). Synthesis of very narrow multilayer graphene nanoribbon with turbostratic stacking. Appl. Phys. Lett..

[37] Lui CH, Liu L, Mak KF, Flynn GW, Heinz TF (2009). Ultraflat graphene. Nature.

[38] Graf D (2007). Spatially resolved raman spectroscopy of single- and few-layer graphene. Nano Lett..

[39] Malard LM, Pimenta MA, Dresselhaus G, Dresselhaus MS (2009). Raman spectroscopy in graphene. Phys. Rep. Rev. Sect. Phys. Lett..

[40] Latil S, Meunier V, Henrard L (2007). Massless fermions in multilayer graphitic systems with misoriented layers: Ab initio calculations and experimental fingerprints. Phys. Rev. B.

[41] Cancado LG (2008). Measuring the degree of stacking order in graphite by Raman spectroscopy. Carbon.

[42] Mucciolo ER, Neto AHC, Lewenkopf CH (2009). Conductance quantization and transport gaps in disordered graphene nanoribbons. Phys. Rev. B.

[43] Wei CP (2019). Turbostratic multilayer graphene synthesis on CVD graphene template toward improving electrical performance. Jpn. J. Appl. Phys..

[44] Negishi R (2019). Turbostratic stacking effect in multilayer graphene on the electrical transport properties. Phys. Status Solidi B-Basic Solid State Phys..

[45] Morozov SV (2008). Giant intrinsic carrier mobilities in graphene and its bilayer. Phys. Rev. Lett..

[46] Uemura K, Ikuta T, Maehashi K (2018). Turbostratic stacked CVD graphene for high-performance devices. Jpn. J. Appl. Phys..

[47] Yu QK (2011). Control and characterization of individual grains and grain boundaries in graphene grown by chemical vapour deposition. Nat. Mater..

[48] Negishi R, Akabori M, Ito T, Watanabe Y, Kobayashi Y (2016). Band-like transport in highly crystalline graphene films from defective graphene oxides. Sci. Rep..

[49] Ambegaokar V, Halperin BI, Langer JS (1971). Hopping conductivity in disordered systems. Phys. Rev. B-Solid State.

[50] Negishi R, Kobayashi Y (2014). Extraordinary suppression of carrier scattering in large area graphene oxide films. Appl. Phys. Lett..

[51] Eda G, Fanchini G, Chhowalla M (2008). Large-area ultrathin films of reduced graphene oxide as a transparent and flexible electronic material. Nat. Nanotechnol..

[52] Ponomarenko LA (2008). Chaotic dirac billiard in graphene quantum dots. Science.

[53] Son YW, Cohen ML, Louie SG (2006). Energy gaps in graphene nanoribbons. Phys. Rev. Lett..

[54] Han MY, Ozyilmaz B, Zhang YB, Kim P (2007). Energy band-gap engineering of graphene nanoribbons. Phys. Rev. Lett..

[55] Nagashio K, Yamashita T, Nishimura T, Kita K, Toriumi A (2011). Electrical transport properties of graphene on SiO2 with specific surface structures. J. Appl. Phys..

[56] Adam S, Hwang EH, Galitski VM, Das Sarma S (2007). A self-consistent theory for graphene transport. Proc. Natl. Acad. Sci. U.S.A..

[57] Miyazaki H (2008). Inter-layer screening length to electric field in thin graphite film. Appl. Phys. Express.

[58] Molitor F (2010). Energy and transport gaps in etched graphene nanoribbons. Semicond. Sci. Technol..

[59] Guinea F (2007). Charge distribution and screening in layered graphene systems. Phys. Rev. B.

[60] Negishi R (2011). Thickness control of graphene overlayer via layer-by-layer growth on graphene templates by chemical vapor deposition. Jpn. J. Appl. Phys..

[61] Negishi R (2011). Layer-by-layer growth of graphene layers on graphene substrates by chemical vapor deposition. Thin Solid Films.

[62] Schwierz F (2010). Graphene transistors. Nat. Nanotechnol..

[63] Negishi R, Ohno Y, Maehashi K, Matsumoto K, Kobayashi Y (2012). Carrier transport properties of the field effect transistors with graphene channel prepared by chemical vapor deposition. Jpn. J. Appl. Phys..

[64] Niimi R (2019). Effect of a protective layer on a carbon nanotube thin film channel in a biosensor device. Jpn. J. Appl. Phys..

[65] Harada N, Awano Y, Sato S, Yokoyama N (2011). Monte Carlo simulation of electron transport in a graphene diode with a linear energy band dispersion. J. Appl. Phys..

[66] Hirai H, Tsuchiya H, Kamakura Y, Mori N, Ogawa M (2014). Electron mobility calculation for graphene on substrates. J. Appl. Phys..

