# Supplementary material for: Crossover point of the field effect transistor and interconnect applications in turbostratic multilayer graphene nanoribbon channel
Source: Sci Rep. 2021 May 13;11:10206. doi: 10.1038/s41598-021-89709-z (PMC8119723; doi:10.1038/s41598-021-89709-z)
Supplement: Supplementary file 1 — Supplementary Information. [file 41598_2021_89709_MOESM1_ESM.pdf]

Supplementary figures

Title:

Crossover point of the field effect transistor and interconnect applications in turbostratic multilayer graphene nanoribbon channel

Ryota Negishi[1,†*], Katsuma Yamamoto[1], Hirofumi Tanaka[2,3], Seyed Ali Mojtahedzadeh[1], Nobuya Mori[1], and Yoshihiro Kobayashi[1]

[1]*Graduate School of Engineering, Osaka University, 2-1 Yamadaoka, Suita, Osaka 565-0871, Japan*

[2]*Research Center for Neuromorphic AI Hardware, Kyushu Institute of Technology2-1 Hibikino, Wakamatsu, Kitakyushu 808-0196, Japan*

[3]*Graduate School of Life Science and System Engineering, Kyushu Institute of Technology, 2-1 Hibikino, Wakamatsu, Kitakyushu 808-0196, Japan*

*Corresponding author; Tel: +81-49-239-1366, FAX: +81-49-239-1366

E-mail address: negishi046@toyo.jp (R. Negishi)

†Present affrication: Faculty of Science and of Engineering, Department of Electrical, Electronics and Communications Engineering, Toyo University, 2100 Kujirai, Kawagoe, Saitama 350-8585, Japan

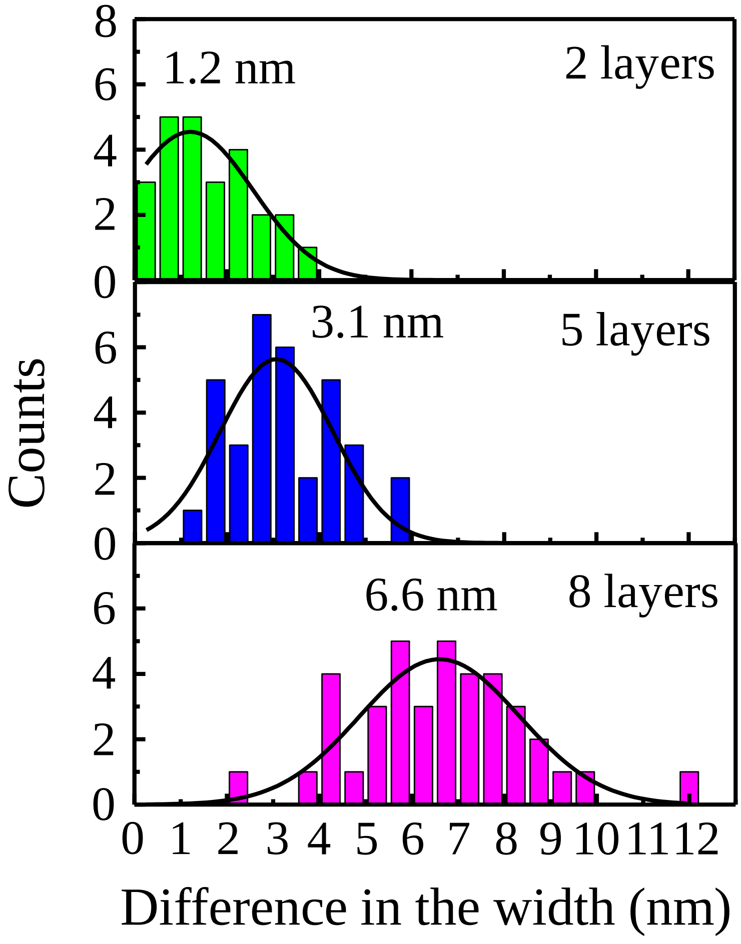

Supplementary Figure 1: ***Width distribution of the multilayer graphene nanoribbons.*** The width is carefully evaluated from the height profiles of GNR using AFM measurements. If the size scale of the observation target material is comparable to the curvature radius of the AFM tip, the target's height profile is strongly affected by the tip shape. We compensate the width of the GNR obtained from the height profiles, assuming that the radius curvature of the AFM tip curvature is 7 nm[1]. The distribution of the GNR widths can be fitted by a Gaussian function. The width distribution obviously increases as the number of layers increases by the CVD growth. This means that the width of GNR slightly expands via a lateral growth from edge sites.

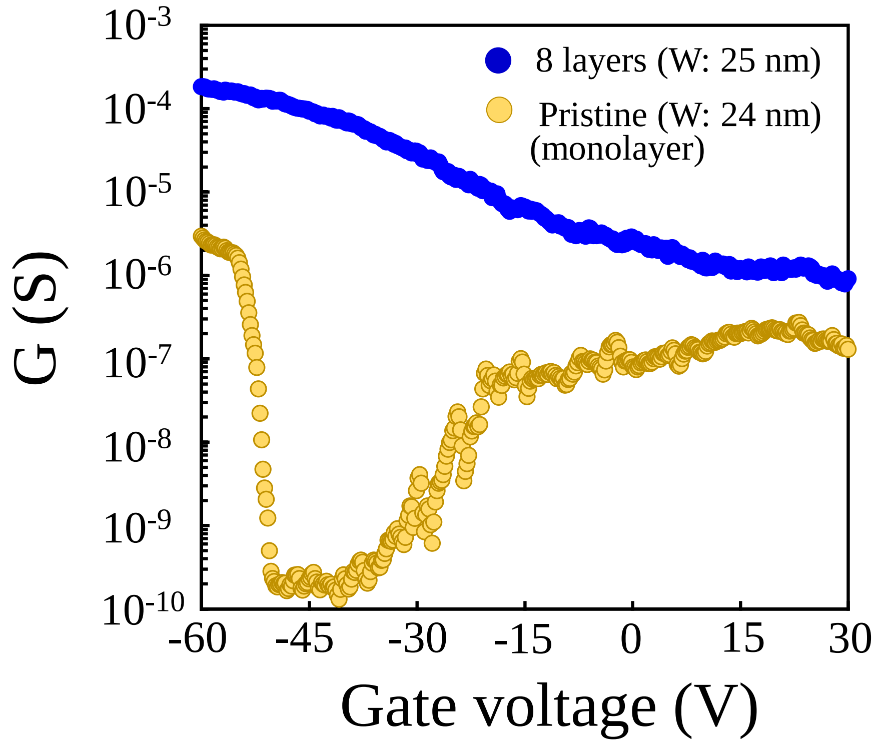

Supplementary Figure 2: ***Transfer characteristics in the pristine monolayer GNR with the width of 24 nm and the grown multilayer with the width of 25 nm.*** Although the width is almost the same between the pristine and multilayer GNRs, only the monolayer pristine graphene shows the OFF state due to the strong suppression of conductance. This is caused by the difference in the number of layers related to the strength of the screening effect.

Supplementary references

1 Negishi, R. *et al.* Synthesis of very narrow multilayer graphene nanoribbon with turbostratic stacking. *Appl. Phys. Lett.* **110**, 201901-4 (2017).
